# Evaluation of Empirical Tropospheric Models Using Satellite-Tracking Tropospheric Wet Delays with Water Vapor Radiometer at Tongji, China

**DOI:** 10.3390/s16020186

**Published:** 2016-02-02

**Authors:** Miaomiao Wang, Bofeng Li

**Affiliations:** College of Surveying and Geo-Informatics, Tongji University, Shanghai 200092, China; 5wmmgps@tongji.edu.cn

**Keywords:** tropospheric delay model, mapping function, water vapor radiometer, kinematic precise point positioning

## Abstract

An empirical tropospheric delay model, together with a mapping function, is commonly used to correct the tropospheric errors in global navigation satellite system (GNSS) processing. As is well-known, the accuracy of tropospheric delay models relies mainly on the correction efficiency for tropospheric wet delays. In this paper, we evaluate the accuracy of three tropospheric delay models, together with five mapping functions in wet delays calculation. The evaluations are conducted by comparing their slant wet delays with those measured by water vapor radiometer based on its satellite-tracking function (collected data with large liquid water path is removed). For all 15 combinations of three tropospheric models and five mapping functions, their accuracies as a function of elevation are statistically analyzed by using nine-day data in two scenarios, with and without meteorological data. The results show that (1) no matter with or without meteorological data, there is no practical difference between mapping functions, *i.e.*, Chao, Ifadis, Vienna Mapping Function 1 (VMF1), Niell Mapping Function (NMF), and MTT Mapping Function (MTT); (2) without meteorological data, the UNB3 is much better than Saastamoinen and Hopfield models, while the Saastamoinen model performed slightly better than the Hopfield model; (3) with meteorological data, the accuracies of all three tropospheric delay models are improved to be comparable, especially for lower elevations. In addition, the kinematic precise point positioning where no parameter is set up for tropospheric delay modification is conducted to further evaluate the performance of tropospheric delay models in positioning accuracy. It is shown that the UNB3 model is best and can achieve about 10 cm accuracy for the N and E coordinate component while 20 cm accuracy for the U coordinate component no matter the meteorological data is available or not. This accuracy can be obtained by the Saastamoinen model only when meteorological data is available, and degraded to 46 cm for the U component if the meteorological data is not available.

## 1. Introduction

When traveling through the atmosphere, global navigation satellite system (GNSS) signals are typically affected by two kinds of error sources; namely, ionosphere and neutral atmosphere. The delays caused by neutral atmosphere are also conventionally referred to as tropospheric delays [1,2]. Although ionospheric delays are usually larger than tropospheric delays in magnitude, they can be basically eliminated by using the so-called ionosphere-free combination with dual- or multi-frequency GNSS signals thanks to their frequency-dependent property. Different from the ionosphere, the troposphere is a non-dispersive medium for microwaves and its delays are frequency-independent [3,4,5]. One can never effectively eliminate its influence similar to ionosphere delay exclusion even with multi-frequency GNSS signals, particularly in observation scenarios with highly spatiotemporal troposphere variations [6]. In geodetic applications, one usually first uses empirical models (e.g., [7,8,9]) to correct tropospheric delays, and then applies parameter estimation, for instance, the piecewise function [10,11] or the random walk process [12,13], to compensate for the residuals. Since most parts of tropospheric delays are corrected by empirical models (about 90%), their correcting efficiency greatly influences geodetic application performance. In other words, the existing high-precision GNSS techniques are limited, to a great extent, by the accuracy of empirical tropospheric correction models, though with those correction models the robustness of troposphere-affected integer ambiguity resolution is expectable in most of the moderate atmospheric scenarios [14].

Tropospheric delay is comprised of hydrostatic and wet delays. Both delays are smallest in the zenith direction and increase approximately proportional to the cosecant of the satellite elevation angle. Hydrostatic delay is highly related to air pressure and temperature. It can reach 2–2.5 m in zenith and has a smooth and slow time-varying characteristic. Hence, hydrostatic delay can be precisely modeled with a few millimeter accuracy or even better with surface meteorological data. Wet delay is usually much smaller than the hydrostatic one. In zenith, the wet delay is only a few centimeters or smaller in arid regions and about 0.35 m in humid regions [5]. However, unlike the hydrostatic delay, the wet delay is mainly dominated by water vapor in the atmosphere and is very complex in spatiotemporal variation [15,16]. Thus, it is rather difficult to accurately model and remove the wet delay even with meteorological data. In this paper, we restrict our scope to evaluating the tropospheric models in wet delay calculation.

In addition to the tropospheric delay model, the mapping function, which is used to describe the dependence of a slant tropospheric delay (STD) on the zenith counterpart, is also very important for wet delay prediction. The mapping function is always a function of the elevation angle of the slant signal. A fair number of mapping functions have been developed by many pioneers in the past two decades, such as the Ifadis [17] and Niell [18] mapping functions, *etc*. Obviously, the empirical wet delay correction performance depends not only on the accuracy of the zenith wet delay model but also the wet mapping function. Unfortunately, water vapor is mainly contained in the near-surface air and has very complicated spatiotemporal variation. As a result, the wet mapping functions have poor accuracy, especially for the scenarios of low elevations.

Precise GNSS applications need to correct the tropospheric delays as accurately as possible, for which one needs to identify an accurate empirical tropospheric model together with a mapping function. In this paper, we will evaluate different empirical tropospheric models together with mapping functions by comparing the model-calculated wet delays with those precise ones measured by water vapor radiometer. The ground-based water vapor radiometer measures sky emission at two or more well-separated frequencies with high temporal resolution. It can scan and track all visible satellites individually and precisely measure the slant wet delays at their azimuths and elevations. Additionally, the kinematic precise point positioning (PPP) in which no tropospheric delay parameter is set up after correction with empirical tropospheric models is conducted for further evaluating these tropospheric models by analyzing their corresponding positioning accuracies.

The paper is organized as follows. In Section 2, we will briefly review some popularly used tropospheric models and mapping functions that will be evaluated. In Section 3, we will introduce how to collect the slant wet delays by the ground-based water vapor radiometer with its satellite tracking function. In Section 4, we will compare the wet delays derived from the empirical models to the water vapor radiometer data. The wet delay errors are statistically analyzed. In Section 5, kinematic precise point positioning with different tropospheric models was conducted for further evaluation. Finally, some concluding remarks are summarized in Section 6.

## 2. Tropospheric Delay Calculation

The total slant tropospheric delay (STD) consists of a slant hydrostatic delay (SHD) and a slant wet delay (SWD), and both of them can be expressed by a relevant zenith tropospheric delay (ZTD) and a mapping function (MF). The total STD is:

STD = SHD + SWD = ZHD × MF_H_ + ZWD × MF_W_ (1)

where ZHD and ZWD are the zenith hydrostatic delay and zenith wet delay, respectively; MF_H_ and MF_W_ are their corresponding MFs. As mentioned, there are many ZTD models and MFs, and the ZHD and MF_H_ are usually very precise. Hence, we focus on evaluating different tropospheric delay models and MF_W_s. Some empirical models, Saastamoinen [7,8], Hopfield [19,20], and UNB3 [21,22], and mapping functions, Chao [23], Ifadis [17], Vienna Mapping Function 1 (VMF1) [24], Niell Mapping Function (NMF) [18], and MTT Mapping Function (MTT) [9], that are popularly used in GNSS positioning applications will be evaluated. Note in Equation (1), the gradient effect used to capture the atmospheric horizontal anisotropy is neglected, since the purpose is to compare different empirical tropospheric delay models.

The Saastamoinen model reads [7,8]:

(2) $$\text{ZTD}=\text{ZHD}+\text{ZWD}=\frac{0.0022768}{1-0.00266\cos(2\varphi )-0.00000028H}\left(P+(\frac{1255}{T}+0.05)e\right)$$

where *P*, *T*, and *e* are the atmospheric pressure in hPa, temperature in Kelvin, and partial water vapor pressure in hPa, respectively; *ϕ* and *H* are the latitude and height of the station in radians and meter, respectively.

The Hopfield model used reads [19,20]:

(3) $$\text{ZTD}=\text{ZHD}+\text{ZWD}=10^{-6}\times (k_{1}\frac{P}{T}\frac{40136+148.72(T-273.16)-H}{5}+(273(k_{2}-k_{1})+k_{3})\frac{e}{T^{2}}\frac{11000-H}{5})$$

where the constants are *k*_1_ = 77.604 K/hPa, *k*_2_ = 64.79 K/hPa, and *k*_3_ = 3.776 × 10^5^ K^2^/hPa.

The UNB3 model used reads [21,22]:

(4) $$\text{ZTD}=\text{ZHD}+\text{ZWD}=\tau_{h}^{z}\cdot \kappa_{h}\cdot P+\tau_{w}^{z}\cdot \kappa_{w}\cdot \frac{e}{T}$$

with

$$\kappa_{h}={\left(1-\frac{\beta H}{T}\right)}^{\frac{g}{R_{d}\beta }}\;,\;\kappa_{w}={\left(1-\frac{\beta H}{T}\right)}^{\frac{\lambda \prime g}{R_{d}\beta }-1}\;,\;\tau_{h}^{z}=\frac{10^{-6}k_{1}R_{d}}{g_{m}}\;,\;\tau_{w}^{z}=\frac{10^{-6}k_{3}^{\prime }R_{d}}{g_{m}\lambda \prime -\beta R_{d}}$$

where *g* = 9.80665 m/s^2^; *R_d_* = 287.054 J/kg/K; $k_{3}^{\prime }$ = 382,000 K^2^/hpa; *g_m_* = 9.784(1 − 2.66 × 10^−3^cos(2*ϕ*) − 2.8 × 10^−7^*H*) m/s^2^; *λ*’ = *λ* + 1. The parameters, *P*, *T*, *e*, *β*, and *λ*, can be calculated by the expression:

(5) $$\xi (\varphi ,doy)=\xi_{avg}(\varphi )-\xi_{amp}(\varphi )\cos(2\pi \frac{doy-28}{365.25})$$

where *ξ* denotes the respective parameter to be computed; the variable *doy* is the day of year. *ξ*_avg_(*ϕ*) and *ξ*_amp_(*ϕ*) are linearly interpolated with the latitude of a station. The interpolation information for meteorological data used in the UNB3 model is shown in Table 1.

**Table 1:** The interpolation information for meteorological data used in the UNB3 model [22].

***ϕ*(°)**	*P* (hpa)	*T* (K)	*e* (hpa)	*β* (K/km)	*λ* (n/a)	*P* (hpa)	*T* (K)	*e* (hpa)	*β* (K/km)	*λ* (n/a)
Average						Amplitude				
15	1013.25	299.65	26.31	6.30	2.77	0.00	0.00	0.00	0.00	0.00
30	1017.25	294.15	21.79	6.05	3.15	−3.75	7.00	8.85	0.25	0.33
45	1015.75	283.15	11.66	5.58	2.57	−2.25	11.00	7.24	0.32	0.46
60	1011.75	272.15	6.78	5.39	1.81	−1.75	15.00	5.36	0.81	0.74
75	1013.00	263.65	4.11	4.53	1.55	−0.50	14.50	3.39	0.62	0.30

The information of the ZWD models and MFWs under investigation is show in Table 2 and Table 3, respectively.

**Table 2:** Information about the three tropospheric delay models that will be evaluated. “M-data” indicates that the meteorological observation will be involved (√) or not (×) in the model; while “Time” indicates whether time is involved (√) or not (×) in the model.

Tropospheric Delay Model	M-Data	Time	Abbreviation
Saastamoinen	√	×	SAAS
Hopfield	√	×	HOPF
UNB3	√	√	UNB3

**Table 3:** Information about the five MFWs that will be evaluated. “M-data” indicates that the meteorological observations will be used (√) or not (×) in MF_W_; while “Time” indicates whether the MFw is a function of time (√) or not (×).

MF_W_	M-Data	Time	Abbreviation
Chao	×	×	Chao
Ifadis	√	×	Ifadis
Vienna Mapping Function 1	×	√	VMF1
Niell Mapping Function	×	√	NMF
MTT Mapping Function	√	×	MTT

With three tropospheric delay models and five wet mapping functions, we obtain 15 SWD combinations of tropospheric models and MF_W_ that can be used to calculate slant wet delays. Since meteorological data can be assimilated by some tropospheric delay models and MF_W_s, in the following experiments, performances of these combinations with, and without, meteorological data are also examined.

## 3. Data Collection

A water vapor radiometer is a meteorological instrument which can be utilized for tropospheric wet delay measuring. It measures thermal radiation from the sky at microwave frequencies, and the air emission is caused by the amounts of water vapor, liquid water, and oxygen in atmosphere [15,25]. The direct measurements of a water vapor radiometer are brightness temperatures, from which wet delays can be derived. Typically, two or more frequencies (normally, one is around 20 GHz and the other is around 30 GHz) are used to separate contributions from water vapor and liquid water in clouds. The water vapor radiometer data has a very high temporal resolution corresponding to a one second sampling rate.

**Figure 1:**
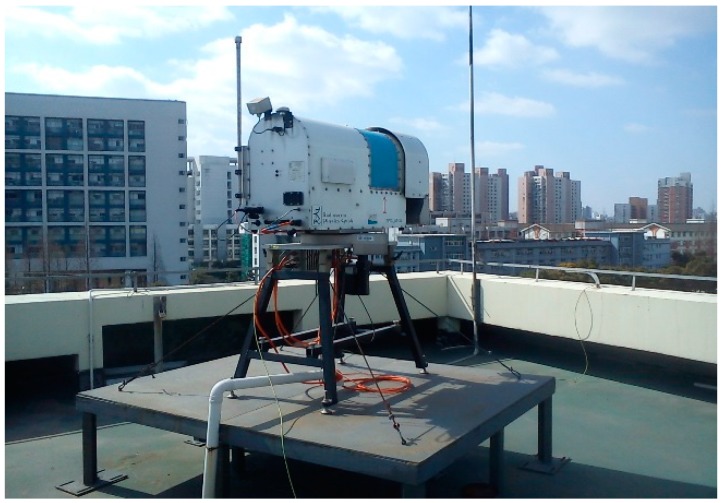
The water vapor radiometer used for data collection on the roof of Cehui building on the Tongji campus (Shanghai, China).

**Figure 2:**
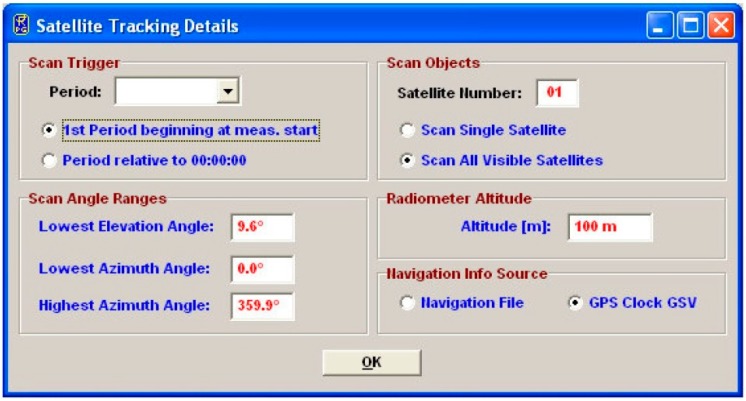
The configuration interface of satellite-tracking function of the used water vapor radiometer. In this configuration, it allows changes to the observation period, the ranges of satellite elevation, and azimuth, the tracking satellite and so on.

The ground-based water vapor radiometer (RPG-LWP manufactured by Radiometer Physics, Meckenheim, Germay) used in our study, as shown in Figure 1, has two frequencies, one is 23.84 GHz and the other is 31.40 GHz. The nominal accuracy of the radiometer is 0.1 kg/m^2^ for integrated water vapor, which is equivalent to 0.1 mm accuracy for precipitable water vapor, and further, about 0.65 mm accuracy for tropospheric wet delay [26] if the atmospheric weighted mean temperature [27] in the transformation coefficient is sufficiently accurate. The radiometer used in this study has a capability of satellite tracking. In Figure 2 the configuration of satellite tracking is displayed. With the provided navigation information of GPS satellites, such as the navigation file, the radiometer can scan the water vapor for a specific satellite or all visible satellites at given elevation and azimuth angle ranges. The satellite tracking function of the radiometer allows measuring slant wet delays of all GPS satellites with a certain sampling interval, such as two minutes.

The satellite-tracked slant wet delays were collected with the water vapor radiometer over the time period of 22–30 November 2014. The elevation cutoff is set to 5° and the azimuth angle ranges from 0° to 360°. Since the water vapor radiometer is weather-sensitive and does not work well in heavy rain or foggy conditions [25], the observations with liquid water path larger than 0.3 mm [28] or 0.7 mm [29] or over 0.5 s (*θ*) kg·m^−2^, where *θ* denotes the elevation angle of the path [30], are discarded. In this study, considering the weather conditions over data collection, and to fully use the observations, we removed the observations only in very extreme weather conditions with liquid water path larger than 1.5 mm, in which cases strong water vapor gradients may have been removed. About 11% of the observations were removed.

The “sky-mapping” water vapor radiometer is “field-of-view”, and its observations are also error-affected. However, the errors of the model-calculated wet delays are larger than those of the radiometer measurements, especially in the low elevation cases. The purpose of this work is to study the difference between some popularly used tropospheric delay models. In the following comparison experiments, the model computed wet delays were compared with the referenced ones measured by the water vapor radiometer. Although the accuracy of different models computed with error-affected radiometer measurements as reference may not be ground truth accuracy, it does not affect the comparison of accuracy difference between different tropospheric delay models.

## 4. Comparison Experiments

As meteorological observations can be employed by some tropospheric models and MFWs, in the following comparison experiments, SWD calculation combinations are evaluated with and without meteorological observations respectively. Taking the radiometer-measured slant wet delays (for each visible GPS satellite at its elevation and azimuth angle) as reference, the differences between wet delays calculated by empirical tropospheric delay models and mapping functions and those slant wet delays measured by radiometer are defined as the wet delay errors of the empirical models. The performance of all empirical tropospheric delay models and mapping functions are statistically analyzed with those wet delay errors.

### 4.1. Standard Atmosphere

**Figure 3:**
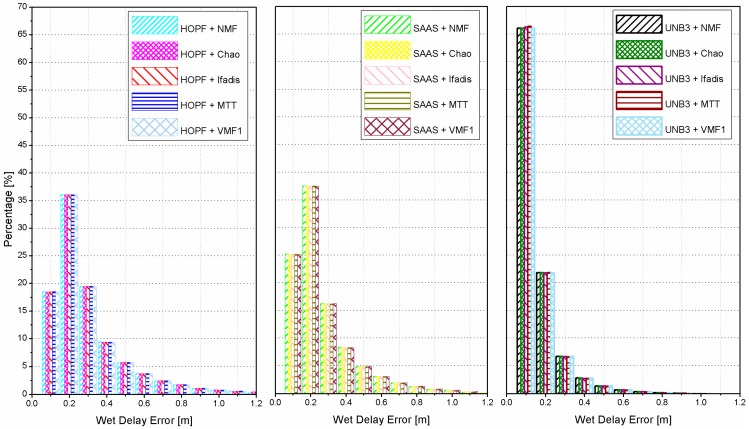
Histograms of absolute wet delay errors derived from all combinations of tropospheric delay models and mapping functions, where the standard atmosphere was applied.

If the meteorological data (atmospheric pressure, temperature, and partial water vapor pressure) is not accessible, one can use the standard atmosphere to compute them, and then calculate the wet delays with empirical models. The histograms of the absolute values of wet delay errors are shown in Figure 3 for all combinations of tropospheric delay models and mapping functions.

The results clearly show that significant statistical differences of wet delay errors are obtained by different tropospheric delay models while statistical differences of wet delay errors by different mapping functions are very minor. The UNB3 model outperforms the SAAS model as well as the HOPF model. Utilizing the UNB3 model, there are about 66% and 88% of the wet delay errors smaller than 0.1 m and 0.2 m, respectively. These values are reduced to about 25% and 62% for the SAAS model, and to only about 18% and 55% for the HOPF model. The most accurate wet delays can be derived by the UNB3 model due to its reasonable representation for meteorological parameters (atmospheric pressure, temperature, partial water vapor pressure, *etc.*). In the UNB3 model, the meteorological parameters of an arbitrary station are interpolated in terms of its geographic position and time (day of year). However, in the HOPF and the SAAS model, the meteorological data used are only related to the station height. Compared with the HOPF model, the delays calculated by the SAAS model are also corrected by the latitude of a station.

**Figure 4:**
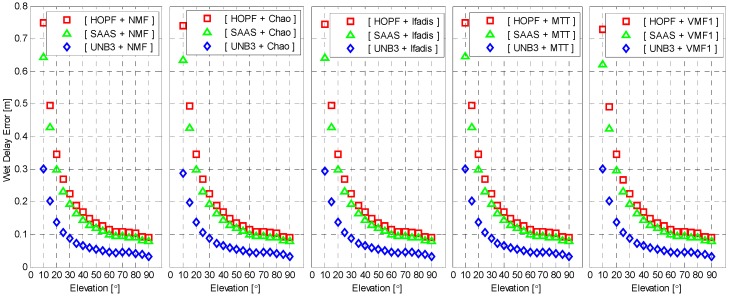
Mean of absolute wet delay errors as function of elevation. The standard atmosphere was applied for wet delay calculation.

To closely examine the performance of different mapping functions, we compute the mean of absolute wet delay errors for each given elevation angle intervals of 5° from 5° to 90°. The result is shown in Figure 4 as function of elevation angles. The results reveal that the wet delay errors are exponentially decreased as the elevation angles increase. The mean wet delay errors are about 0.3 m and 0.03 m, with respect to elevation angles of 10° and 90° for the UNB3 model, about 0.65 m and 0.08 m for the SAAS model, and about 0.75 m and 0.09 m for the HOPF model. Again, it is clear that the UNB3 model outperforms the other two models for all elevations although the difference becomes smaller for high elevations. The overall performance of SAAS and HOPF models coincides very well but, for lower elevations, the SAAS model outperforms the HOPF model significantly.

**Figure 5:**
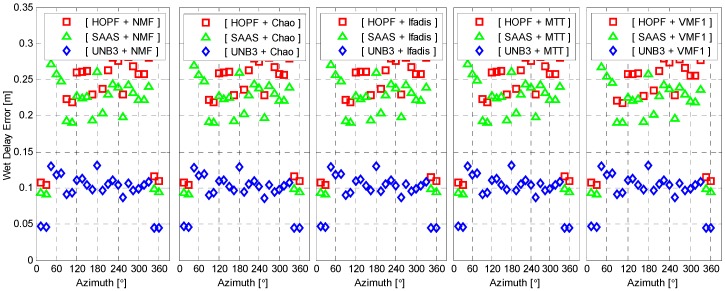
Mean of the absolute wet delay errors as a function of azimuth. The standard atmosphere was applied for wet delay calculation.

All of the used empirical tropospheric delay models assume that the tropospheric delays are elevation-dependent, but azimuth-independent, *i.e.*, they are under the assumption of atmospheric horizontal isotropy. We simply examine how large the variation of wet delay errors can be over the azimuth angles. Figure 5 illustrates the mean of the absolute wet delay errors for each azimuth angle interval of 15° from 0° to 360°. Obviously, the variations of wet delay errors are very small between different azimuth angles, and they are indeed azimuth-independent (the large liquid water path has been excluded, in which cases the strong gradients of water vapor could be eliminated).

**Figure 6:**
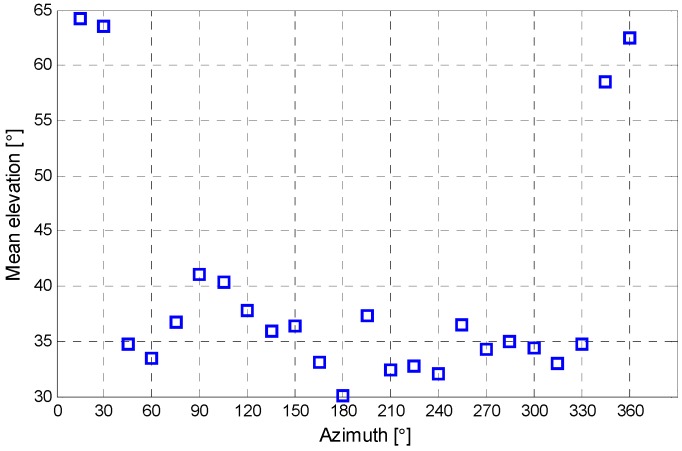
Mean elevation in each azimuth angle interval.

The mean wet delay errors in the azimuth angle interval of about 330°–30° are smaller than those in other azimuth angle intervals. To study this, the mean elevation angle in each azimuth angle interval is shown in Figure 6. As shown, mean elevation angles in azimuth angle interval of about 330°–30° are higher than those in other azimuth angle intervals. The higher mean elevation angles contribute to the smaller wet delay errors, which are also shown in Figure 4.

### 4.2. Meteorological Observations

**Figure 7:**
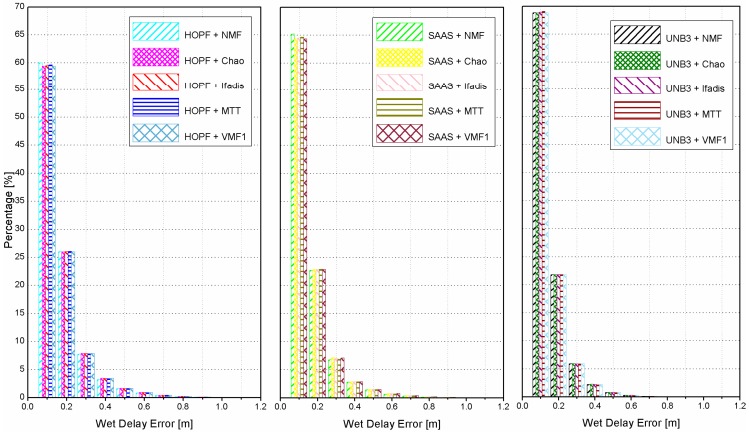
Histograms of absolute wet delay errors derived from all combinations of tropospheric delay models and mapping functions, where the real meteorological data were applied.

In this experiment, instead of standard atmosphere, the meteorological measurements continuously collected also by the water vapor radiometer are employed to compute wet delays by empirical tropospheric delay models and mapping functions. Similar to Figure 3, the histograms of absolute wet delay errors are shown in Figure 7 for all combinations of tropospheric models and mapping functions. When the meteorological observations are employed, the performances become very close for different tropospheric delay models and mapping functions. The percentage of absolute wet delay errors smaller than 0.2 m is improved from 55% and 62% for HOPF and SAAS models both to 85%. However, for the UNB3 model, the improvement is marginal since the reasonable meteorological parameters can already be obtained by interpolation of the model itself.

**Figure 8:**
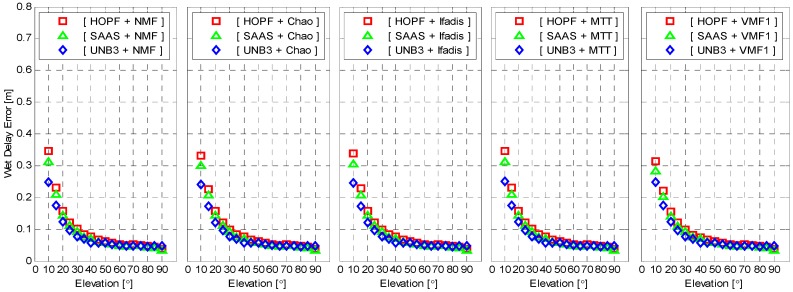
Mean of absolute wet delay errors as function of elevation. The meteorological measurement was applied for the wet delay calculation.

**Figure 9:**
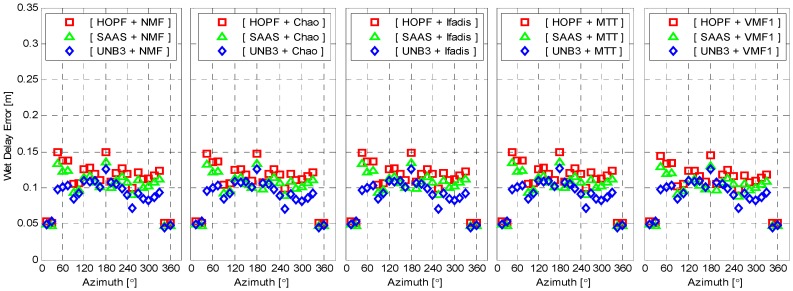
Mean of absolute wet delay errors as a function of azimuth. The real meteorological data was applied for wet delay calculation.

The wet delay errors calculated from meteorological observations are presented in Figure 8 as a function of elevation. It is still elevation-dependent. However, the wet delay errors with real meteorological observations are significantly reduced for all elevations. The maximum of wet delay errors is reduced to about 0.3 m from 0.65 m and 0.75 m, with respect to SAAS and HOPF models. All combinations of tropospheric delay models and mapping functions now coincide very well. Similar to Figure 5, the wet delay errors with meteorological observations are presented in Figure 9 as a function of azimuth angle. The behavior of wet delay errors is completely similar to those in Figure 5 except that the wet delay errors in Figure 9, overall, become smaller.

Compared with meteorological data derived from standard atmosphere or other atmospheric models (e.g., [31,32]), the observed meteorological data has a better capability for reflecting the real atmosphere situation. The comparisons of empirical tropospheric models with, and without, meteorological observations also reveals that the meteorological observations are important to improve the accuracy of wet delays with empirical tropospheric delay models and mapping functions, especially for the SAAS and HOPF models.

By statistically analyzing the absolute values of wet delay errors, the magnitude of them are clearly shown. The mean and standard deviation of all wet delay errors of empirical tropospheric delay models with and without meteorological data are shown in Table 4.

The same conclusions as above can be drawn from the comparison between the mean and standard deviation of wet delay errors of different empirical tropospheric delay models with, and without, meteorological measurements.

**Table 4:** The mean and standard deviation (std) of wet delay errors of empirical tropospheric delay models (in cm).

	Standard Atmosphere						Meteorological Observations					
	HOPF		SAAS		UNB3		HOPF		SAAS		UNB3	
	Mean	std	Mean	std	Mean	std	Mean	std	Mean	std	Mean	std
NMF	−24.56	20.88	−21.04	18.91	−9.34	11.07	−10.90	11.68	−9.72	11.27	−3.39	11.85
Chao	−24.49	20.72	−20.95	18.74	−9.20	10.90	−10.77	11.48	−9.58	11.09	−3.22	11.78
Ifadis	−24.53	20.81	−21.00	18.84	−9.27	11.00	−10.84	11.59	−9.66	11.19	−3.32	11.82
MTT	−24.58	20.91	−21.06	18.95	−9.34	11.08	−10.90	11.68	−9.72	11.27	−3.42	11.86
VMF1	−24.37	20.50	−20.81	18.50	−9.34	11.07	−10.57	11.22	−9.38	10.85	−3.39	11.85

### 4.3. Satellite-Specific Wet Delay Errors

**Figure 10:**
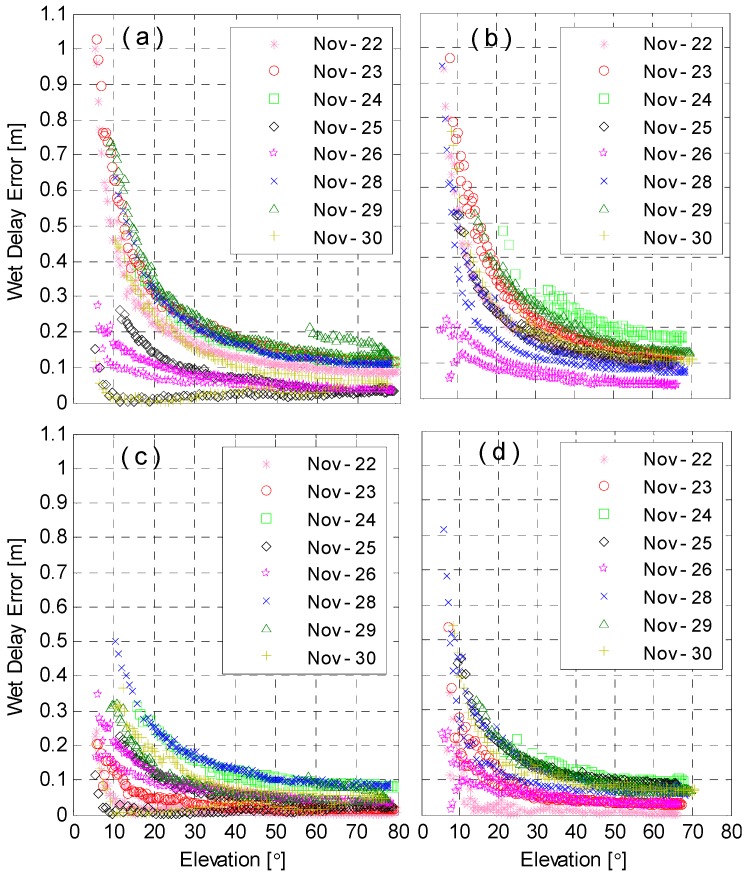
The absolute wet delay errors calculated with SAAS and NMF for satellites PRN 8 and 13 without and with meteorological observations. The subplots (**a**,**b**) denote the results without meteorological observations for PRN 8 and 13; while (**c**,**d**) denote the results with meteorological observations for these satellites.

The above statistical analyses are obtained based on the wet delays over multiple days with different, but relatively dry, atmospheric conditions. The satellite-tracking function of the radiometer allows us to have a closer analysis of the performance of different tropospheric delay models and mapping functions in those different atmospheric conditions. With satellites PRN 8 and 13 as examples, wet delay errors with, and without, meteorological observations are computed by the tropospheric delay model of the SAAS and mapping function of NMF over the period of 22–30 November 2014 are computed. The absolute wet delay errors are shown in Figure 10 as a function of elevation angles for all days.

For PRN 8 and 13, the wet delay errors generally increase exponentially with decreasing elevations, and in different days, they are significantly different. Wet delay errors computed with meteorological observations are, overall, much smaller than those without them. Without meteorological observations, the maximal wet delay error is about 1 m for both PRN 8 and 13, and the minimal elevation of wet delay errors smaller than 0.2 m is about 45°. However, with real meteorological observations, the maximal wet delay error reduced to about 0.7 m and the minimal elevation for errors smaller than 0.2 m is reduced to about 25°. These results also confirm the importance of meteorological observations for wet delays calculation.

## 5. Kinematic PPP with Different ZTD Models

The above analyses are based on directly comparing the wet delays from the empirical tropospheric models and the radiometer measurements. To further illustrate performances of these empirical tropospheric models, the kinematic PPP (Precise Point Positioning) experiments were carried out with nine-day (22–30 November 2014) dual-frequency GPS data collected by a Trimble NetR9 receiver (at a fixed station) with a sampling interval of 15 s. Meantime, the meteorological data at the receiver site with a sampling interval of 30 s was collected by Vaisala’s PTU303 as well. The PPP software used in the experiment was self-developed by the GNSS group, Tongji University (Shanghai, China). The software has been intensively tested internally and externally showing stable, and better (or at least comparable), results. The software is also very powerful since it has implemented versatile functions for scientific research. For the comparison purpose, we first conducted a static PPP computation by using nine-day data to obtain very precise station coordinates, which will be used as reference. In this computation, the ionosphere-free combinations are applied. Additionally, correction with the UNB3 model for tropospheric errors, the ZTD parameters are set up to absorb the residuals.

In PPP processing, the cut-off elevation was set to 5° and 15 s meteorological data were linearly interpolated from 30 s data. The final products of precise satellite orbits and clocks of 30 s from IGS (International GNSS service) were used. The ionosphere-free combination was applied to eliminate ionosphere delays, and the Earth tide, ocean tide, relativity, phase windup, and other conventional effects were all corrected. Note, in the tropospheric correction, we applied the UNB3 model with meteorological data to calculate the ZHD, and then used NMF to map it into slant directions. Since the aforementioned comparisons have shown that the HOPF is worse than the SAAS and UNB3, we applied only the SAAS and UNB3 models to calculate the ZWD corrections with, and without, meteorological data, respectively. Then, the NMF mapping function was applied to obtain the slant wet delays. If the residual tropospheric delay was treated as time-dependent parameters, the quite similar (even equivalent) positioning results will be obtained no matter which tropospheric delay model was applied. For comparison, we did not set up ZTD parameters anymore after correcting for tropospheric delays with empirical models. As a result, the residual tropospheric errors will remain in the PPP solutions, and reflect the performance of the empirical tropospheric models.

**Figure 11:**
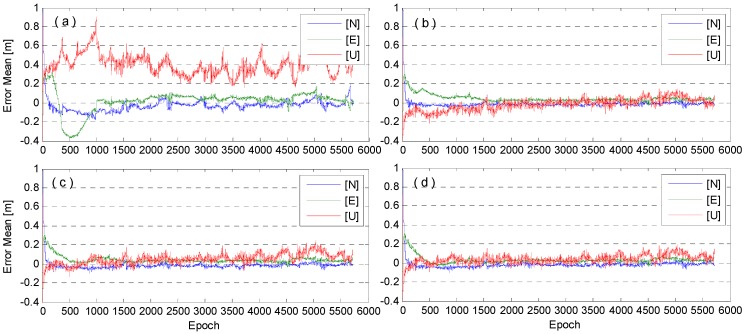
The positioning errors of kinematic PPP, where N, E, and U denote the North, East, and Up components. The subplots (**a**,**c**) denote the results of the SAAS and UNB3 where no meteorological data were used; and the subplots (**b**,**d**) denote the results of the SAAS and UNB3 where meteorological data were used.

The positioning errors of kinematic PPP solutions for 22 November are shown in Figure 11, from which the following conclusions are drawn. First of all, without meteorological data, the UNB3 model significantly outperforms the SAAS model. The UNB3 model leads to a shorter convergence time and smaller fluctuation, especially for the Up direction, due to its strong correlation with troposphere [6]. When the ground based meteorological data are used, the results with UNB3 and SAAS models are both improved, particularly in the case of the SAAS model. Nevertheless, the SAAS approach still needs relatively more convergence time. Since the UNB3 model without meteorological data has already very small positioning errors, the improvement when introducing real meteorological data is very slight.

**Figure 12:**
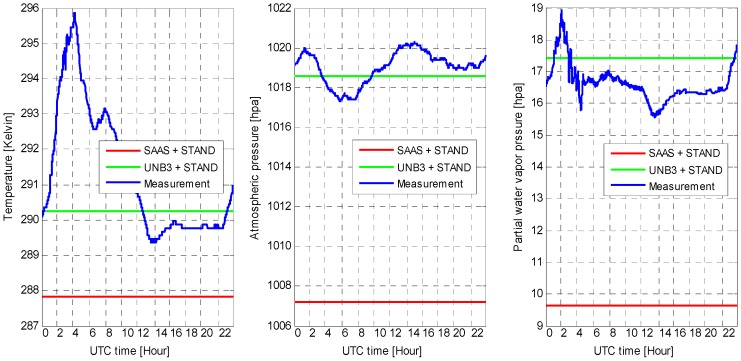
The temperature, atmospheric pressure, and partial water vapor pressure used to calculate the tropospheric delay on 22 November. The ″SAAS + STAND″ and ″UNB3 + STAND″ are denoted as the modeled meteorological data used in the SAAS and UNB3 model. ″Measurement″ is denoted as the observed meteorological data.

The meteorological data used to calculate the tropospheric delays (hydrostatic delay and wet delay) on 22 November are shown in Figure 12. As shown, the meteorological observations are time-variant, while the modeled ones are not. Additionally, compared with the meteorological data used in the SAAS model, the data used in the UNB3 model is more close to the observed data.

**Figure 13:**
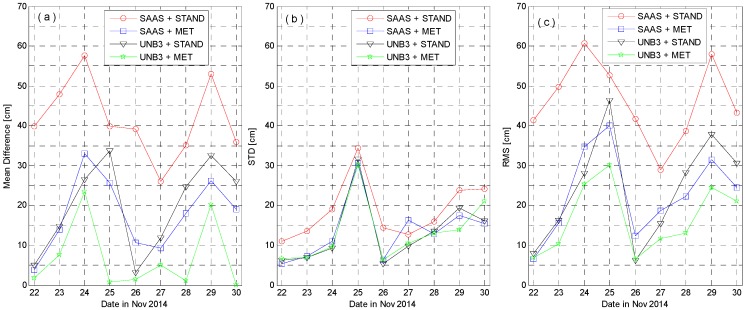
Statistics of kinematic PPP positioning errors in the Up coordinate component for nine-day data. The subplots, (**a**–**c**) denote the results of mean difference (its absolute value), STD, and RMS of positioning errors.

To statistically evaluate the positioning errors with different tropospheric delay models, we conduct the kinematic PPP computations for nine-day data from 22–30 November 2014. The daily statistics, the mean difference (its absolute value), the standard deviation (STD), and the root mean squares (RMS) of positioning errors are computed. Since the troposphere delays affect mainly the Up component, we only show the statistical results for the Up component in Figure 13. The standard deviations on 25 November are larger than those on the other days, while the absolute mean differences are not. This is probably due to the poor situation with larger observation noises. The mean RMS of the three coordinate components (North, East, and Up) over nine-day kinematic PPP solutions for four types of empirical tropospheric delay models is presented in Table 5.

**Table 5:** The mean RMS of nine-day kinematic PPP solutions for four types of empirical tropospheric delay models. “MET” and “STAND” mean wet delays are calculated with and without meteorological data, respectively.

	SAAS + STAND (cm)	SAAS + MET (cm)	UNB3 + STAND (cm)	UNB3 + MET (cm)
N	10.74	9.26	9.54	8.96
E	12.76	8.66	8.81	8.07
U	46.14	22.94	24.12	16.65

The mean error, STD, and RMS of kinematic PPP results vary daily from ten to several tens of centimeters. Without the observed meteorological data, the SAAS model yields to about 46 cm accuracy in the Up component. It means that in this case, the residual tropospheric errors are still significant and they should be properly compensated, for instance, by setting up a ZTD parameter, so as to get accurate positioning results. When the real meteorological data is introduced, the correction capability of the SAAS model is dramatically improved to about 9 cm for the North and Earth, while about 23 cm for the Up component. However, the UNB3 model without meteorological data can even obtain results comparable to the SAAS model with meteorological data. The results with the UNB3 model can be further improved by using the real meteorological data.

## 6. Conclusions

The effects of atmosphere on GNSS signals and their applications are considerable, and the delays caused by the neutral atmosphere (often referred to as the troposphere) are the main limit of high-precision applications, besides the delays caused by the ionosphere. Different to the methods for ionospheric delay eliminating, the empirical tropospheric delay model is commonly employed to correct the tropospheric errors in pre-processing of GNSS observations. Therefore, the tropospheric delay models are very important for GNSS applications. In this paper 15 combinations of three empirical tropospheric models (SAAS, HOPF, UNB3) and five mapping functions (Chao, Ifadis, VMF1, NMF, MTT) were evaluated by statistically comparing their computed wet delays with those measured by a water vapor radiometer. Two application scenarios identified by whether using meteorological measurement or not were examined for all 15 combinations. Finally, with the SAAS and UNB3 models as example, we further evaluated these empirical models for wet delay correction by analyzing their kinematic PPP solutions, where no ZTD parameter was set up after correcting tropospheric delays with empirical models. Based on the comprehensive statistical analysis, the following conclusions are obtained.

No matter the real meteorological data is available or not, there is no practical statistical difference between mapping functions (*i.e.*, Chao, Ifadis, VMF1, NMF and MTT) in computing the slant tropospheric wet delays. However, when the real meteorological data is not available, the tropospheric delay models (*i.e.*, SAAS, HOPF, and UNB3) are significantly different in computing the tropospheric wet delays. The model precision is ordered by UNB3, SAAS, and HOPF, where the UNB3 model is most precise because of its proper expression of meteorological parameters. When the real meteorological data is available, all three tropospheric delay models are comparable. The model error is about 0.3 m for elevation angles lower than 10°, and over all elevations. The model error is smaller than 0.1 m for at least 60% of the test cases, while smaller than 0.2 m for 85%.

The kinematic PPP results reassure that the UNB3 model is the most accurate. Even without real meteorological data, its results are comparable with the SAAS with real meteorological data. The result indicates that if decimeter accuracy solution (10 cm for North and East, and 20 cm for Up) is required, one can use the UNB3 model with or without meteorological data or the SAAS model with meteorological data. However, if more accurate results are required, one has to compensate the residual tropospheric errors by a proper way, for instance, by setting up additional ZTD parameters in the PPP model.
